# Microsurgery-aided in-situ force probing reveals extensibility and viscoelastic properties of individual stress fibers

**DOI:** 10.1038/srep23722

**Published:** 2016-03-30

**Authors:** Céline Labouesse, Chiara Gabella, Jean-Jacques Meister, Benoît Vianay, Alexander B. Verkhovsky

**Affiliations:** 1 Laboratory of Cell Biophysics, Ecole Polytechnique Fédérale de Lausanne, Lausanne, Switzerland

## Abstract

Actin-myosin filament bundles (stress fibers) are critical for tension generation and cell shape, but their mechanical properties are difficult to access. Here we propose a novel approach to probe individual peripheral stress fibers in living cells through a microsurgically generated opening in the cytoplasm. By applying large deformations with a soft cantilever we were able to fully characterize the mechanical response of the fibers and evaluate their tension, extensibility, elastic and viscous properties.

Stress fibers - bundles of actin and myosin II filaments associated with many accessory proteins – are major tension-generating and tension-bearing structures in adherent cells^1^. Despite the large number of studies, there is still no comprehensive understanding of the mechanical properties of and of the mechanisms of tension generation by stress fibers^2^,^3^,^4^,^5^. Fiber tension has been attributed largely to the activity of the motor protein myosin II^1^,^5^, but many studies have also suggested the existence of a passive elastic component, the origin of which is not clear^6^,^7^. *In vitro* reconstitution of actin-myosin fibers from purified proteins illuminated the mechanisms of tension-generation by disordered filament assemblies^8^,^9^,^10^, but this approach has still a way to go to reconstruct the entire complexity of native stress fibers. Stress fibers isolated from extracted cells displayed remarkable extensibility under rigor conditions^3^,^11^, but were partially destabilized in the presence of ATP^12^. On the other hand, mechanical probing of native stress fibers in the living cells is limited by the geometry of the cell and difficult to interpret because of interactions with the surrounding cytoplasm and the substrate^7^,^13^,^14^. So far, the main approach to induce large deformations in order to investigate viscoelastic response of the fiber *in situ* was laser ablation^5^,^15^,^16^, which has only been done as a one-time probing: even if the fiber recovers after ablation, this most likely happens as full remodeling rather than reassembly of the same fiber^5^. Extraction and isolation of the individual fibers from the cells offer more control and degrees of freedom for their probing, but remove them from their natural cytosolic environment^2^,^3^,^11^,^17^.

Here we present an original approach to study the mechanics of peripheral stress fiber in living cells. Peripheral stress fibers are responsible for the geometry of non-protrusive edges of the cell. Several previous studies used peripheral fibers bridging non-adhesive gaps on patterned substrates as a biophysical model for mechanical analysis^7^,^14^,^18^,^19^. Peripheral fibers show typical stress fiber protein content with alpha-actinin and myosin II, organized in an intermittent but non-periodic fashion^7^, although myosin II isoform composition and regulatory pathways may differ between peripheral and central stress fibers^5^,^20^,^21^,^22^. Here we combine microsurgery with a soft force probing to isolate the peripheral fibers and to test their response to deformation. This method presents the advantage of a simple and well-defined geometry, and offers the possibility of virtually unlimited reversible deformation allowing evaluating the full range of viscoelastic fiber properties.

For experiments, cells were plated on 5 μm high elevated adhesive patterns to make peripheral fibers accessible from the side without risking contacting the substrate with the probe^7^,^14^. We then used glass pipettes to create an opening in the cytoplasm just behind a peripheral fiber, so that the fiber became isolated from the cytoplasm along most of its length, but remained attached to adhesions at its extremities (Fig. 1A,B). After severing the connection with the cytoplasm, the fiber became straight (Supplementary Figure 1). This is consistent with previous studies that attributed curvature of peripheral fibers to the balance of line tension within the fiber and surface tension from the membrane and cytoplasmic actin network acting normally to the fiber^7^,^19^. Isolation of the fiber eliminates this normal tension thereby leading to a zero curvature, *i.e.* a straight fiber. Straightening occurred over approximately the same time-scale (a few seconds) as relaxation observed during subsequent mechanical probing (see below), suggesting that the same viscoelastic properties were also at play during fiber isolation. Microscopic observation after microsurgery revealed that in most of the cases the cell remained attached to the pattern and retained a protrusive activity at its edges, including edges of the opening which led eventually to the closure of the hole within hours, thus allowing ample time for probing of the fiber. We observed two types of hole repair: a closure by protrusion from the main cell body (Supplementary Figure 1B) and a closure by sliding on the fiber towards the cell body (Supplementary Figure 1C). This activity indicated that microsurgery was not deleterious to the cell. In some cases, when the hole was in a region close to the nucleus or where the cytoplasm was too thick, we observed an obvious deleterious effect on the cell in the form of shrinking or detachment from the substrate: in this case the experiments weren’t pursued.

Notable advantages of this setup are that the fiber is straight, making it easier to analyze its deformation^17^,^23^,^24^ and that inserting a probe between the cell and the fiber allows to stretch it outwards to achieve virtually unlimited deformation. The fiber was stably connected to the cell through adhesions, making this configuration sustainable and compatible with repeated mechanical stimulations and pharmacological perturbations.

The fiber was deformed by applying a displacement perpendicular to the fiber axis with a soft cantilever^14^ at a controlled speed in two phases: a ramp (∼5 μm/s), over a course of ∼20 μm, then a plateau during which the stage was kept still while the fiber relaxed (Fig. 1C–E and Supplementary Movie). Tension of the fiber was obtained from the cantilever force using force balance analysis, similarly to previous studies^17^,^24^ (see Materials and Methods and Fig. 1F). In contrast to these studies, our approach allowed applying large deformation to individual peripheral stress fibers in a simple linear geometry. We typically probed the same fiber up to 5 times for approximately 10 seconds each, over a period of 15–30 minutes. Since the exposure time of the fiber to the external force was short, we did not expect force-induced remodeling, as observed in a recent study, also using elevated patterned substrates^25^.

The tension of semi-isolated fibers under no external force (termed hereafter pre-tension *T*_*0*_) was obtained by extrapolation from the tension-deformation curve at zero deformation, (Fig. 2A-inset). We have also measured tension in the peripheral fibers before their isolation using small inward deformation (compression) with the same cantilever^14^. We obtained pre-tension values in semi-isolated fibers of 1–18 nN, similar to previous reports on extracted stress-fibers^2^,^17^. However, tension measured by laterally compressing peripheral fibers prior to isolation was approximately 10 times higher^14^. Compression of the isolated peripheral fibers yielded the same low tension values as their stretching, indicating that the difference in tension between isolated and non-isolated fibers was due to the isolation and not to the direction of force application (Fig. 2A). A reason for higher apparent values and greater variability in the pre-tension of the peripheral fibers before isolation is probably that the deformation by the probe may engage to a variable extent the supporting fibers immediately behind the edge and the adjacent actin cortex, so that in fact the ensemble of the closely spaced fibers was probed. This interpretation is supported by fluorescence imaging of actin fibers (Fig. 2B and Supplementary Figure 1A) showing the presence of a supporting meshwork of fibers close to the non-isolated peripheral fiber that are eliminated during isolation. On the other hand, image of the isolated fiber demonstrates that the structural integrity of the edge fiber itself was maintained during the microsurgery and subsequent probing (Fig. 2B), but the fluorescence intensity profile shows a sharper and lower peak compared to the fiber not subjected to isolation (Fig. 2C). We conclude that isolation allows for tension measurements in very thin bundles, providing an approximation of an individual stress fiber. This could be a first step to understand the properties of the whole fiber ensemble. In the following, we chose to apply only outward deformations (stretching followed by relaxation), as they allow larger deformations (up to 82% strain), which are useful to measure the elastic properties of fibers with high precision.

To simultaneously assess elastic and viscous response we analyzed the time course of the resistive force *F*_*clv*_ and of the transverse fiber displacement *e* (Fig. 3). Given the linear trend of fiber tension with deformation during the stretching phase (Fig. 2A-inset), a spring constant could be extracted from the slope of that curve. However, if there were viscous dissipation in the system, this would lead to an overestimation of the spring constant. We indeed observed significant tension relaxation (~18% of the maximum tension value) when the stage was kept still (see Fig. 1D, panels II–III), indicating that the contribution of viscosity could not be neglected. During the relaxation phase the transverse fiber displacement increased exponentially, allowing to extract the system’s (fiber + cantilever) characteristic relaxation timescale *τ* (Fig. 3A-inset), in average 2.3 +/−0.23 seconds. To account for the observed time courses of *e* and *F*_*clv*_ during the stretching and relaxation phases, we described the full mechanical response of the fiber using a modified standard-linear solid model (Fig. 3B). The model is fully characterized by 4 parameters, namely pre-tension *T*_*0*_, spring constants *k*_*1*_ and *k*_*2*,_ and viscosity coefficient *η*. *T*_*0*_ and *τ* are independently measured (see Figs 2A and 3A); *k*_*2*_ and *η* are not independent, but related through the relaxation time scale *τ*. We find the two remaining independent parameters *k*_*1*_ and *k*_*2*_ by fitting the experimental curves of *e(t)* and *F*_*clv*_*(t)* (Fig. 3C,D and Table 1) and minimizing the aggregated error (see Materials and Methods for detailed fitting procedure).

This modified standard-linear model well accounted for the observed viscoelastic behavior (Fig. 3); Spring constant *k*_*1*_ and fiber pre-tension *T*_*0*_ describe fiber elasticity by accounting for most of the resistive force during stretching. The values of these parameters and the corresponding fiber stiffness *EA* (*EA* = *k*_*1*_*d*) values (up to 200 nN), were consistent with previous reports^2^,^3^,^11^. The contribution of the second spring *k*_*2*_ is minor at the moderate elongation velocities used in our setup, as evidenced by the small amplitudes of relaxation. In this model, *k*_*2*_ is essentially an auxiliary element to tune the force due to viscosity *η*. These parameters describe the mechanical properties of the fiber as a whole, however, it is not known if these properties are uniform along the fiber length. Images indicate a significant heterogeneity in thickness and F-actin fluorescence intensity along the fiber length, which may be an indication of the heterogeneity of the mechanical properties. Further studies will be necessary to investigate how specific mechanical properties are associated with specific domains of the fiber.

The most striking feature of fiber behavior under external force was their ability to extend up to twice their initial length without breaking, and to fully recover back to their rest length. Matsui *et al.*^11^ reported a similar high extensibility for extracted fibers but it was not clear if the extension was fully elastic and reversible. Here, we found that tension increased largely linearly over the whole range of extension. This high extensibility cannot be explained by the extensibility of any one individual constituent of the fiber (myosin motors, actin cross-linkers or actin filaments), but could in principle be accounted for by the telescopic sliding of actin filaments connected to each other by motors or cross-linkers. However, it is unclear how a sliding mechanism would lead to a tension increasing with extension. High fiber extensibility is also compatible with the fluidity of the plasma membrane, and membrane tension could contribute to the measured fiber tension. However, its contribution is expected to be very small since the tension necessary to pull cytoskeleton-free membrane tethers is one to two orders of magnitude lower than the measured fiber tensions^26^, and the tension in membrane tethers is independent of tether length. Elastic behavior of the fibers could be due to non-muscle titin^27^, as suggested by Matsui and co-workers^11^, which is a very extensible, elastic protein, or another yet unknown elastic component.

Even if the myosin II alone cannot explain fiber extensibility, myosin activity could affect the spring constant of the fiber by modifying the cross-linking arrangement. To investigate myosin II’s contribution to fiber tension and spring constant, we conducted tension measurements while attenuating myosin activity with ROCK inhibitor Y27632 (10 μM). We compared the values after 40 min of ROCK inhibition to the values before the drug was added, and have observed that fiber pre-tension *T*_*0*_ decreased by a factor of ~2 (Fig. 3E,F and Table 1), while in control conditions, no such drop was observed (data not shown). This result demonstrates that semi-isolated fibers retained myosin activity and its regulation by ROCK and is consistent with previous report in which pre-tension was attributed to motor^5^,^15^. Our experiments, however, do not allow to determine if the measured T_0_ values were entirely due to active tension resulting from myosin activity, or partly active and partly elastic. The latter could be the case if the actual rest length of the fiber was smaller than its measured length. T_0_ therefore gives an upper bound of active tension rather than its exact value. There was no significant effect of Y27632 on the spring constants *k*_*1*_ and *k*_*2*_, or on *η*. Notably, when released, stretched fibers shortened back to their initial length as in control conditions, even after 40 minutes of myosin inhibition, indicating that high extensibility and elastic behavior are independent of myosin contractility (Supplementary Figure 3).

Further work is required to better understand how specific mechanical properties of actin-myosin fibers are related to their molecular composition and structural arrangement and to identify specific mechanisms and components responsible for the parameters measured in our study. The setup described here is well suited for such investigations, allowing for multiple mechanical measurements under various conditions, from each of which information on the tension, elasticity and the viscosity of stress fibers could be extracted. This could be combined with fluorescent microscopy to reveal the dynamics of specific proteins during mechanical probing, as well as with functional approaches such as RNAi silencing or optogenetics to selectively (de)activate motors or crosslinking capacity. Since our approach allows in principle the control of fiber dimensions, it may also reveal how the mechanical properties scale with the size of the fiber and illuminate the internal substructure and possible heterogeneity of the fibers. These results could be compared and contrasted to those obtained using reconstituted actin-myosin networks with varying concentrations of cross-linkers and motors.

## Materials and Methods

### Cell culture and myosin inhibition

We used an immortalized rat embryonic fibroblast cell line (REF-52) for our experiments. Cells were cultured in Dulbecco’s modified Eagle Medium (DMEM High Glucose, Gibco, Grand Island, NY, USA) supplemented with 1% penicillin, streptomycin, L-glutamine and 10% fetal calf serum (Gibco). Cells were detached with 0.02% EDTA in PBS and 0.25% trypsin (Gibco), seeded on the elevated patterned substrates and allowed to spread for at least 6 hours prior to experiments. Experiments were performed in bicarbonate-free medium (Leibovitz’s L15, Gibco) at room temperature. ROCK pathway of myosin activation Y27632 was used at 10 μM in Leibovitz’s L15 medium and added after stress fiber isolation. Measurements were performed on a same fiber before myosin inhibition and after 40 minutes of drug incubation for at least 20 minutes, to detect any change of behavior due to myosin inhibition.

### Immunostaining

Before fixation, cells were washed with serum-free medium. Cells were first fixed with 4% paraformaldehyde in PBS and permeabilized with 0.2% Triton-X100 for 5 minutes. Primary antibody rabbit anti-myosin IIb (Cell Signaling, Beverly, MA, USA) and phalloidin conjugated to Alexa-Fluor 568 (Molecular Probes) were both incubated for 1 h, then rinsed with PBS.

### Confocal imaging

Confocal stacks of stained cells were obtained on a Zeiss LSM700 upright confocal microscope. Z-slices were taken every 600 nm over 12 μm, and a maximal projection was then obtained from the stack.

### Elevated structured substrate fabrication and patterning

Elevated structured substrates were fabricated using customized soft lithography technique as previously described^14^. Briefly, a PDMS mold was obtained by spin-coating a PDMS elastomer (Sylgard 184, Dow Corning Corporation: 10:1 base/curing agent ratio) on a 5 μm-deep silicon master to a thickness of 10 μm, then a round glass coverslip previously treated with sodium hydroxide solution to promote adhesion was put in contact with the PDMS substrate. Finally, the coverslip with the resulting PDMS replica was carefully unmolded after bake at 100 °C during 30 minutes. This generated elevated platforms on which the cells were later seeded. PDMS substrate was treated with UV (254 nm, 16 W) during 30 minutes to promote protein adhesion and subsequently patterned with 10 μg/ml fibronectin (Human Plasma purified, Millipore, Switzerland) and 10 μg/ml Alexa Fluor 647 conjugated fibrinogen. For that, the adhesive protein solution in PBS was previously adsorbed on a glass coverslip pre-treated for 1 minute with an oxygen plasma (50 W RF power) then used as the protein stamp which was put into contact with the PDMS during 2 minutes. The PDMS substrate was then soaked in 2% of Pluronic F-127 in de-ionized water during 1 hour for backfilling, then extensively rinsed with PBS. Thus, only the top surface of the platforms was coated with fibronectin and allowed for cell adhesion, while the space between the platforms and the platform walls were not adhesive. Finally, substrate was incubated during 1 hour with culture medium at 37 °C and 5% CO_2_ before cell seeding.

The geometry of the adhesive patterns were designed to have proper cell spreading according to average cell size and to provide sufficient space in between adhesive branches (‘S’,‘E’ and ‘H’ shapes), so as to have long peripheral fibers bordering thin sheets of suspended cytoplasm with the nucleus being sufficiently far in order not to interfere with the microsurgery.

### Micro-fabrication of SU-8 cantilevers

SU-8 ultra-soft cantilevers were prepared as previously described^14^. Briefly, two layers of SU-8 epoxy (GM1060, Gersteltec SA) were successively coated, soft-baked, exposed then post-exposure baked on a double-side polished silicon wafer, then the assembly was developed and finally stored in cleanroom environment. The first 5 μm SU-8 layer included cantilevers and support designs, and the second 50 μm SU-8 layer included only the support design. The silicon wafer was previously prepared with alignment markers on its backside and with a sputtered 200 nm-thick layer of titan/tungsten (W:Ti 10%) following by a deposition of 800 nm-thick sacrificial layer of aluminum on its frontside for adhesion and further detachment. Finally, probes were detached using anodic dissolution of the aluminum layer in a saline solution with a platinum counter electrode. For the purpose of this study, squared section cantilevers (5 × 5 μm^2^) were used, with spring constants ranging from 1.5 to 6.25 nN/μm.

### Microsurgery

**Figure 1:**
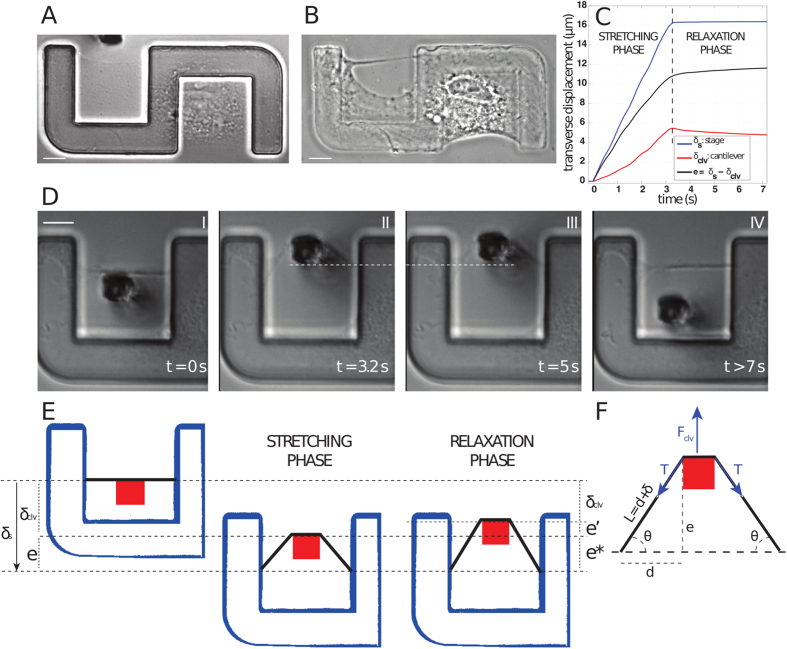
Probing semi-isolated fiber with soft cantilever. (**A**) Cell spread on an elevated S-shaped PDMS pattern. A thin cytoplasmic sheet is bridging the gap between two adhesive sides of the pattern (left side of the cell). (**B**) Phase contrast image of the cell after microsurgery: the fiber is isolated from the cytoplasm. (**C**) Time course of the deformation: *δ*_*s*_ is the imposed stage displacement (blue – top curve), *δ*_*clv*_ is the cantilever displacement (red – bottom curve), *e* is the transverse fiber displacement (see 1F), calculated as the difference between *δ*_*s*_ and *δ*_*clv*_ (black – middle curve). The two phases of deformation are called “STRETCHING PHASE” (constant stage velocity) and “RELAXATION PHASE” (zero stage velocity). (**D**) Snapshots of the experiment in the frame of reference of the substrate. The motion of the stage and cantilever correspond to the displacements represented in 1C. (I) The cantilever is placed between the fiber and the rest of the cell; (II) then the stage is moved, so that the cantilever pulls the fiber outward (stretching phase); (III) the stage is then kept still to let the fiber relax (relaxation phase) and finally (IV) the probe is retracted and the fiber recovers its initial straight configuration. The white dashed line represents the position of the cantilever at the end of stretching phase (II), to highlight the additional small deformation of the fiber in (III). (**E**) Cartoon of the experiment in the laboratory frame of reference. The adhesive pattern is shown in blue, the cross-section of the cantilever in red and the probed fiber in black. Displacements are slightly exaggerated. *e*^∗^ corresponds to the transverse displacement at the end of the traction phase, *e*′ represents additional elongation during the relaxation phase. (**F**) Geometry of the system and parameter: a fiber of initial half-length *d* is pulled over a transverse distance *e* with a cantilever force *F*_*clv*_. The resulting half-length of the elongated fiber is 
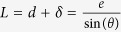
 (*δ*: fiber elongation, *θ*: the projection angle on the initial fiber axis, resisting tension is 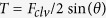). For A, B and D, scale bar is 10 μm.

A glass capillary (Femtotips, Eppendorf AG, Hamburg, Germany) with opening diameter of 0.2 μm to 0.5 μm was mounted on a manual micro-manipulator (Leica, Wetzlar, Germany) and used to perform microsurgery on cells. Isolation of the fiber through microsurgery was successfully performed on cells that had a thin stretch of cytoplasm between the branches of adhesive pattern (Fig. 1A,B). In these cases, the hole in the cytoplasm enlarged spontaneously after an initial small cut, and closed back only over long periods (~hours), thus allowing sufficient time for mechanical probing of the isolated fiber. However, if adjacent focal adhesions were disrupted, injury and fiber manipulation led to the progressive detachment of cells from the patterns, in which case the experiments were not pursued.

### Microscope experimental setup and image analysis

Experiments were performed on an inverted ZEISS Axiovert 200M system with a 40× Plan-Neofluar, air objective (N.A. 0.75). Two three-axis micromanipulators (NMN-25 mounted on top of MX-1, Narishige Scientific Instruments Lab.) were used to position the cantilever. The cantilever holder was kept above the sample and the beam hanged vertically in the field of view of the microscope, free to move. A motorized 3-axis sample stage (MS-2000, Applied Scientific Instrumentation, with NanoDrive controller, Mad City Labs Inc.) was used to move the cells towards the cantilever. Stage motion was controlled by a custom script written in MATLAB^®^ (The MathWorks Inc., MA, USA). Images were acquired using a CoolSnap HQ2 camera (Photometrics, AZ, USA) through the ultra-fast acquisition streaming mode in Metamorph (Molecular Devices, CA, USA).

Cantilever was placed into the hole immediately after surgery, as soon as the hole was sufficiently large to accommodate it (~few minutes, see Supplementary Figure 1C). That was done to block the beginning of the process of resealing, which eventually occurred in some hours. Stage displacement was triggered as soon as the cantilever position was adjusted to the fiber midpoint and the setup stabilized. We controlled that the cantilever motion was perpendicular to the axis of the fiber; if we observed a significant lateral motion during fiber stretching, indicating a major imbalance between both sides of the fiber, the results were discarded. After each successful trial, we removed the cantilever and waited for the fiber to return to its straight position (~a few seconds) before the fiber was stretched again. Total time of force application for one fiber typically did not exceed ~1 min.

Stage displacement (*δ*_*s*_) and cantilever displacement (*δ*_*clv*_) were separately determined using a slice alignment plugin in ImageJ (NIH, Bethesda, Maryland, USA, http://imagej.nih.gov/ij/), as previously described^14^. Acquisition frequencies needed to be of at least 10 Hz to have enough sampling frequency for the deformation velocity of 5 μm/s. Isolated stress fiber half-length *d* was manually measured on image after isolation. The average length of the fiber probed was 28.4 μm +/−3.6 μm (s.d.) with minimal length 22 μm and maximum length 35 μm.

For non-isolated stress fibers, the experimental and analysis procedures follow^14^.

### Data analysis and model fitting: Standard Linear Solid Model with active term

We refers to Fig. 4 for the geometry and the force balance of the system.

The actual deformation rate depended on the relative stiffness of the stress fiber compared to the cantilever stiffness. The stress fiber’s transverse displacement *e* and resistive force *F*_*clv*_ were inferred from the cantilever deflection *δ*_*clv*_ and the stage position *δ*_*s*_ (eq. 1, 2 and Fig. 1C):


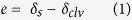



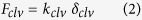


*k*_*clv*_ is the known spring constant of the cantilever, ranging between 1.53–6.25 nN/μm in our experiments. Since we take into account fiber viscosity and the actual timing of the deformation while fitting the data, the stiffness of the cantilever is not expected to affect the parameters extracted from the analysis. Moreover even with the softest cantilever, the displacement of the fiber was usually larger than that of the cantilever (e.g., see Fig. 1C), so using a stiffer cantilever would not affect the speed and the extent of deformation dramatically. We did not observe any systematic differences between fibers probed with cantilevers of different stiffness.

The force on the cantilever *F*_*clv*_ is balanced by the tension *T* in the two fiber segments on either side of the cantilever, projected onto the displacement axis *F*_*clv*_ = 2*T* sin(*θ*) (eq. 3) (Fig. 4A). We assume that the cantilever contacts the fiber in its middle, such that both segments are of equal length. In the experiments we tried to place the cantilever as symmetrical as possible but we cannot exclude that position was slightly off-centered. However, a small asymmetry between the two sides of the fiber is not expected to strongly affect the measurements, because the increase of tension on one half of the fiber would nearly compensate its decrease on the other half. Assuming a linear elastic fiber one could estimate that positioning of the cantilever so that one side of the fiber is 1.5 times longer than the other would only lead to a difference in the tension measurement by about 4% with respect to the perfectly symmetric positioning for the same transverse displacement of the fiber.

After each stimulation, the cantilever was retracted and the fiber was let recover its initial straight configuration, which allowed repeated measurements over periods up to one hour (Supplementary Figure 2).

The evolutions of *e(t)* and *F*_*clv*_*(t)* were then fitted with a modified standard-linear model, which consists of three different parallel modules: a spring with constant *k*_*1*_, a Maxwell viscoelastic module of a spring (*k*_*2*_) in series with a dashpot (*η*), and a motor unit accounting for the pre-tension *T*_*0*_ (Fig. 4B). In principle, solid relaxation could be described by a simple Kelvin-Voigt model. However, in this model the amplitude and the timescale of relaxation are not independent, so that small relaxation amplitudes are incompatible with the long time courses observed in our experiments. Adding a third element in series to the dashpot, as in the standard-linear model, decouples time and amplitude, and allows for a good fit (Fig. 3A,B).

Fitting of the model were performed by adjusting for 2 parameters *k*_*1*_ and *k*_*2*_, as described below.

The main equation describing the system is derived from the force balance using the projection of the tension in the stress fiber onto the transverse axis (along which the cantilever is displaced), as shown on Fig. 4. t* is the moment when the motion of the stage is halted, *δ*_s_* is the position of the stage at that t*, i.e. the maximal elongation reached during the stretching phase.

From the system geometry and the force balance, the evolution of the transversal elongation *e(t)* is described by the implicit differential equation (eq. 4):


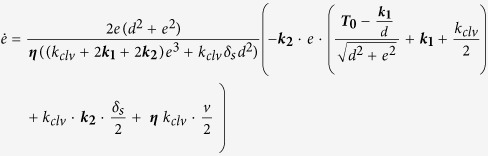


**Figure 2:**
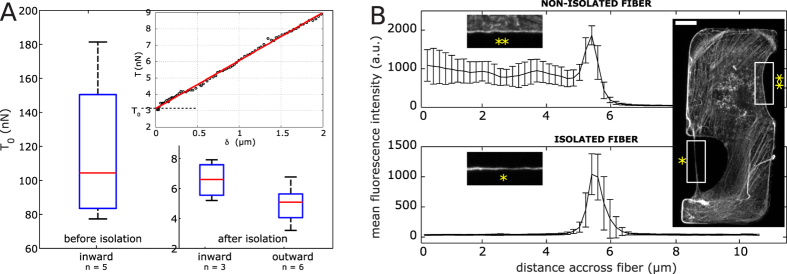
Fiber tension before and after isolation. (**A**) Pre-tension values of non-isolated fibers (“inward before isolation”) compared to isolated fibers deformed inward (“inward after isolation”) and outward (“outward after isolation”). Number of measured fibers for each case (*n*) is indicated, each fiber has been tested in at least two of the three conditions. For each boxplot, the central red line is the median, the edges of the box are the 25th and 75th percentiles and the whiskers (black dashed lines) extend to the most extreme data points not considered outliers. Inset shows a typical force-deformation curve of isolated fiber *T(δ)* where *T* is fiber tension and *δ* is fiber longitudinal elongation. This graph allows extracting the value of pre-tension *T*_*0*_: circles are the experimental values, red line is the linear trend of the data points and *T*_*0*_ is the value of *T* at zero-elongation, i.e. the y-intercept of the graph. (**B**) Fluorescence intensity profiles of the F-actin in non-isolated (^∗∗^upper graph) and isolated (^∗^lower graph) fibers of the cell shown in inset, stained with phalloidin-AlexaFluor568. Profiles are taken transversally to the fibers and averaged along the length (ROIs shown by boxes in the inset), bars show s.d. Fluorescence image was obtained by a maximal projection of a confocal stack, voxel size was 0.20 × 0.20 × 0.60 μm. Bar is 10 μm.

**Figure 3:**
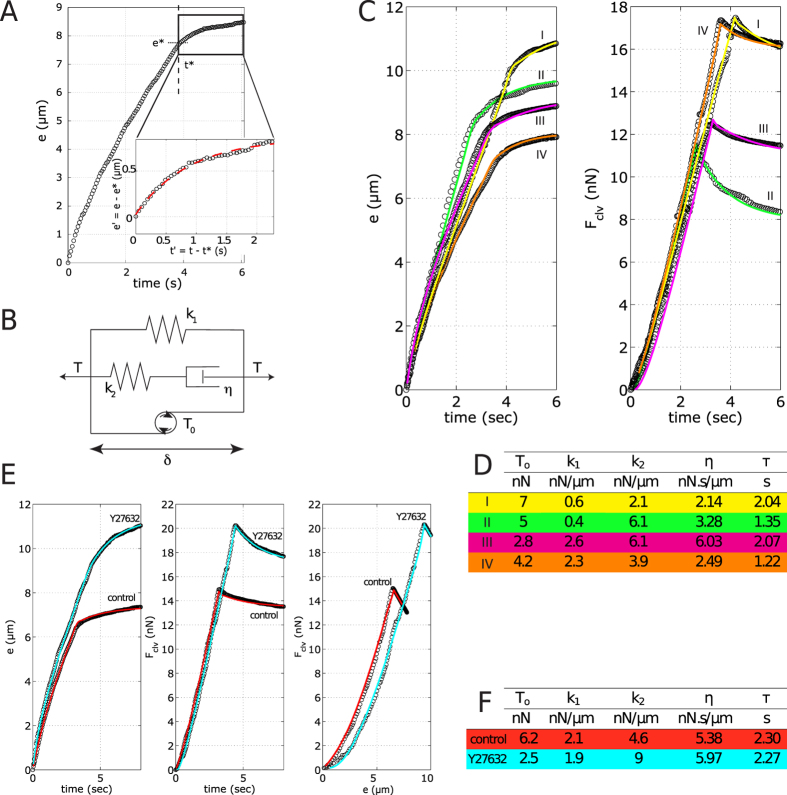
Mechanical model of the isolated fiber. (**A**) Time evolution of the fiber transverse displacement during deformation. Black dashed lines show the time when the motion of the stage is halted, allowing fiber relaxation. Inset magnification of the viscoelastic relaxation *e*′ = *e* − e* in function of *t*′ = *t* − t*. e* is the transverse displacement at the end of the stretching phase and t* is the time when the stage is halted. Red dashed line shows fit of the additional deformation with the exponential function 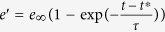, which allows the extraction of the characteristic time *τ*. (**B**) Mechanical model of the fiber. The fiber with tension *T* subjected to elongation *δ* is approximated by a standard linear solid model (SLS) that has 3 elements in parallel: a primary spring of constant *k*_*1*_, a secondary spring (*k*_*2*_) in series with a dashpot (*η*) and an active element corresponding to pre-tension (*T*_*0*_). (**C**) Time evolution of the transverse displacement (left) and the cantilever measured force (right) during deformation of four fibers. Circles indicate experimental values and colored solid lines represent the best fit to the SLS model. (**D**) Parameters of the SLS model for the curves shown in (**C**). Corresponding curves are color-coded. (**E**) Time evolutions of the transverse displacement (left) and the cantilever measured forces (center) during deformation of the same fiber before (red) and after myosin inhibition (cyan). The force-deformation graph (right) illustrates that after myosin inhibition, a lower cantilever force (*F*_*clv*_) is needed to achieve the same deformation (*e*). Circles indicate experimental values and colored solid lines represent the best fit to the SLS model. Corresponding graphs for *T* vs *δ* are found in Supplementary Figure 2, at *t* = *0* (control) and *t* = *51* (Y27632). (**F**) Parameters of the SLS model for the curved shown in (**E**). Corresponding curves are color-coded.

This equation presents four unknown parameters (in bold), which represent the mechanical elements of the system. *v*_*0*_ is the nominal stage velocity (here 5 μm/s) and *k*_*clv*_ is the known cantilever spring constant. We extract the unknown parameters through the following sequential steps:

a. *T*_*0*_ is the so-called pre-tension, i.e. tension of the fiber at zero elongation. Value is extracted from the force-deformation curve, as the y-intercept (see Fig. 2A).

b. Additional elongation *e*′ = *e* − e* of relaxation (at *t* > t*) shows an exponential trend (see Fig. 3A-inset). Fitting it by the equation 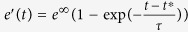, the characteristic time *τ* can be obtained. *e*^*∞*^ corresponds to the plateau value of the elongation, *i.e.* the maximal additional elongation reached. The goodness of fit gave an average R^2^ ~ 0.96.

c. *η* and k_2_ are mutually dependen*t* through *τ* as:


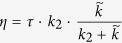


with


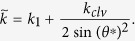


**Figure 4:**
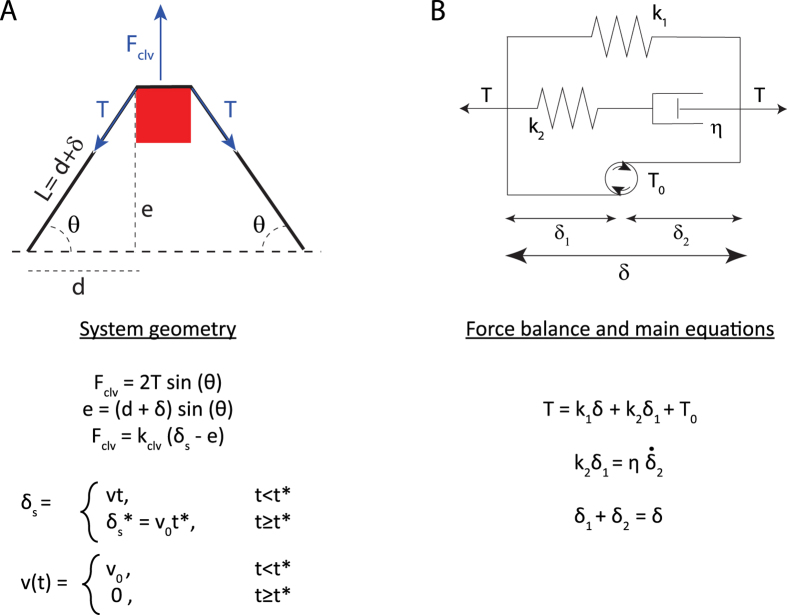
System geometry (**A**) and force balance (**B**) used for deriving the system equation.

*η* is obtained from *k*_*2*_, considering that the angle θ* during the relaxation phase is constant (see Fig. 4) (justified by the fact that the additional elongation is small compared to the e*).

d. *k*_*1*_ and *k*_*2*_ are chosen to fit best the experimental data, by looping on two successive ranges of parameters, with step of 2 nN/μm followed by 0.1 nN/μm. Upper limit of *k*_*1*_ is fixed as for a pure elastic regime: 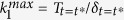. For each couple (*k*_*1*_, *k*_*2*_), (eq. 4) is solved using the MATLAB integrated differential equation solver, *ode45*. Best fit is given by the minimal error from experimental and fit data, calculated as 
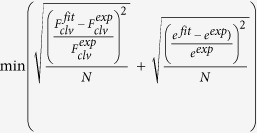
, *N* is the number of points of the fitting interval.

Parameter results are shown in Table 1.

## Additional Information

**How to cite this article**: Labouesse, C. *et al.* Microsurgery-aided in-situ force probing reveals extensibility and viscoelastic properties of individual stress fibers. *Sci. Rep.* **6**, 23722; doi: 10.1038/srep23722 (2016).

## Supplementary Material

Supplementary Information

Supplementary Movie

## Figures and Tables

**Table 1:** Mechanical parameters of semi-isolated stress fibers.

		*T*_*0*_	*k*_*1*_	*k*_*2*_	*η*
CTRL (n = 13)	mean values +/− s.d.	7.44 +/− 5.69 nN	3.23 +/− 2.74 nN/μm	10.94 +/− 10.75 nN/μm	7.85 +/− 6.74 nN.s/μm
Y27632 (n = 3)	mean Y27/CTRL ratio +/− s.d.	0.58 +/− 0.28	1.19 +/− 0.55	0.83 +/− 0.66	0.85 +/− 0.24

Values are mean +/− s.d. First line shows averaged values for *n* = *13* fibers in control conditions (parameter values for each fiber were averaged over up to 5 subsequent pulls). Second line shows the relative changes of the parameters after myosin inhibition calculated as the ratios of the (averaged) parameters measured on a fiber after 40 minutes of drug incubation to the (averaged) parameters measured on the same fiber before drug application (values averaged over *n* = *3* fibers).

## References

[b1] Burridge K. & Wittchen E. S. The tension mounts: Stress fibers as force-generating mechanotransducers. J. Cell Biol. 200, 9–19 (2013). 23295347 10.1083/jcb.201210090 PMC3542796

[b2] Deguchi S., Ohashi T. & Sato M. Evaluation of tension in actin bundle of endothelial cells based on preexisting strain and tensile properties measurements. Mol. Cell. Biomech. 2, 125–133 (2005). 16708474

[b3] Deguchi S., Ohashi T. & Sato M. Tensile properties of single stress fibers isolated from cultured vascular smooth muscle cells. J. Biomech. 39, 2603–2610 (2006). 16216252 10.1016/j.jbiomech.2005.08.026

[b4] Kassianidou E. & Kumar S. A biomechanical perspective on stress fiber structure and function. Biochim. Biophys. Acta-Mol. Cell Res, 10.1016/j.bbamcr.2015.04.006 (2015). PMC4589434 25896524

[b5] Tanner K., Boudreau A., Bissell M. J. & Kumar S. Dissecting regional variations in stress fiber mechanics in living cells with laser nanosurgery. Biophys. J. 99, 2775–83 (2010). 21044574 10.1016/j.bpj.2010.08.071 PMC2965957

[b6] Albert P. J. & Schwarz U. S. Dynamics of Cell Shape and Forces on Micropatterned Substrates Predicted by a Cellular Potts Model. Biophys. J. 106, 2340–2352 (2014). 24896113 10.1016/j.bpj.2014.04.036 PMC4052361

[b7] Labouesse C., Verkhovsky A. B., Meister J.-J., Gabella C. & Vianay B. Cell Shape Dynamics Reveal Balance of Elasticity and Contractility in Peripheral Arcs. Biophys. J. 108, 2437–2447 (2015). 25992722 10.1016/j.bpj.2015.04.005 PMC4457271

[b8] Reymann A. *et al.* Actin Network Architecture Can Determine Myosin Motor Activity. Science (80-). 336, 1310–1314 (2012). 10.1126/science.1221708 PMC3649007 22679097

[b9] Thoresen T., Lenz M. & Gardel M. L. Reconstitution of contractile actomyosin bundles. Biophys. J. 100, 2698–705 (2011). 21641315 10.1016/j.bpj.2011.04.031 PMC3117186

[b10] Thoresen T., Lenz M. & Gardel M. L. Thick filament length and isoform composition determine self-organized contractile units in actomyosin bundles. Biophys. J. 104, 655–65 (2013). 23442916 10.1016/j.bpj.2012.12.042 PMC3566465

[b11] Matsui T. S., Sato M. & Deguchi S. High extensibility of stress fibers revealed by *in vitro* micromanipulation with fluorescence imaging. Biochem. Biophys. Res. Commun. 434, 444–8 (2013). 23583399 10.1016/j.bbrc.2013.03.093

[b12] Matsui T. S., Ito K., Kaunas R., Sato M. & Deguchi S. Actin stress fibers are at a tipping point between conventional shortening and rapid disassembly at physiological levels of MgATP. Biochem. Biophys. Res. Commun. 395, 301–306 (2010). 20353757 10.1016/j.bbrc.2010.03.150

[b13] Lu L., Oswald S. J., Ngu H. & Yin F. C.-P. Mechanical properties of actin stress fibers in living cells. Biophys. J. 95, 6060–71 (2008). 18820238 10.1529/biophysj.108.133462 PMC2599828

[b14] Piacentini N., Verkhovsky A. B., Gabella C., Meister J.-J. & Vianay B. Ultra-soft cantilevers and 3-D micro-patterned substrates for contractile bundle tension measurement in living cells. Lab Chip 14, 2539–47 (2014). 24867825 10.1039/c4lc00188e

[b15] Colombelli J. *et al.* Mechanosensing in actin stress fibers revealed by a close correlation between force and protein localization. J. Cell Sci. 122, 1665–79 (2009). 19401336 10.1242/jcs.042986

[b16] Kumar S. *et al.* Viscoelastic retraction of single living stress fibers and its impact on cell shape, cytoskeletal organization, and extracellular matrix mechanics. Biophys. J. 90, 3762–3773 (2006). 16500961 10.1529/biophysj.105.071506 PMC1440757

[b17] Sugita S., Adachi T., Ueki Y. & Sato M. A novel method for measuring tension generated in stress fibers by applying external forces. Biophys. J. 101, 53–60 (2011). 21723814 10.1016/j.bpj.2011.05.046 PMC3127176

[b18] Théry M., Pépin A., Dressaire E., Chen Y. & Bornens M. Cell distribution of stress fibres in response to the geometry of the adhesive environment. Cell Motil. Cytoskeleton 63, 341–55 (2006). 16550544 10.1002/cm.20126

[b19] Bischofs I., Schmidt S. & Schwarz U. S. Effect of Adhesion Geometry and Rigidity on Cellular Force Distributions. Phys. Rev. Lett. 103, 1–4 (2009). 10.1103/PhysRevLett.103.048101 19659402

[b20] Chang C.-W. & Kumar S. Vinculin tension distributions of individual stress fibers within cell-matrix adhesions. J. Cell Sci. 126, 3021–30 (2013). 23687380 10.1242/jcs.119032 PMC3711198

[b21] Katoh K., Kano Y., Amano M., Kaibuchi K. & Fujiwara K. Stress fiber organization regulated by MLCK and Rho-kinase in cultured human fibroblasts. Am. J. Physiol. Cell. Physiol. 280, 1669–1679 (2011). 10.1152/ajpcell.2001.280.6.C1669 11350763

[b22] Chang C.-W. & Kumar S. Differential Contributions of Nonmuscle Myosin II Isoforms and Functional Domains to Stress Fiber Mechanics. Sci. Rep. 5, 13736 (2015). 26336830 10.1038/srep13736 PMC4559901

[b23] Bernal R., Pullarkat P. & Melo F. Mechanical Properties of Axons. Phys. Rev. Lett. 99, 018301 (2007). 17678192 10.1103/PhysRevLett.99.018301

[b24] Hakari T. *et al.* Nonlinear displacement of ventral stress fibers under externally applied lateral force by an atomic force microscope. Cytoskeleton (Hoboken). 68, 628–38 (2011). 21976314 10.1002/cm.20537

[b25] Scheiwe A. C., Frank S. C., Autenrieth T. J., Bastmeyer M. & Wegener M. Subcellular stretch-induced cytoskeletal response of single fibroblasts within 3D designer scaffolds. Biomaterials 44, 186–194 (2015). 25617137 10.1016/j.biomaterials.2014.12.018

[b26] Gabella C. *et al.* Contact angle at the leading edge controls cell protrusion rate. Curr. Biol. 24, 1126–1132 (2014). 24794299 10.1016/j.cub.2014.03.050

[b27] Cavnar P. J., Olenych S. G. & Keller T. C. S. Molecular identification and localization of cellular titin, a novel titin isoform in the fibroblast stress fiber. Cell Motil. Cytoskeleton 64, 418–433 (2007). 17366640 10.1002/cm.20193

