# Supplementary material for: Microsurgery-aided in-situ force probing reveals extensibility and viscoelastic properties of individual stress fibers
Source: Sci Rep. 2016 Mar 30;6:23722. doi: 10.1038/srep23722 (PMC4812326; doi:10.1038/srep23722)
Supplement: Supplementary Information [file srep23722-s1.pdf]

Microsurgery-aided in-situ force probing reveals extensibility and viscoelastic properties of individual stress fibers

Céline Labouesse[1,*], Chiara Gabella[1,*], Jean-Jacques Meister[1], Benoît Vianay[1,‡] and Alexander B. Verkhovsky[1,‡]

* These authors contributed equally to this work

‡ These authors jointly supervised this work

[1.] Laboratory of Cell Biophysics, Ecole Polytechnique Fédérale de Lausanne, Lausanne, Switzerland

Supplementary Information

Supplementary Figure 1. Cell morphology before and after microsurgery.

Supplementary Figure 2. Repetitive measurements of tension during the extension phase on the same stress fiber before and after the addition of Y27362.

Supplementary Figure 3. Stress fibers maintain visco-elastic properties upon myosin II inhibition.

Supplementary Movie. Manipulation of a semi-isolated bundle in a cell on an elevated substrate. The cantilever is placed in the empty space created by micro-surgery and then the microscope stage is moved to the right, so that the bundle is pulled outwards resulting in a significant elongation (stretching phase). Stage is then halted for a few seconds and the bundle is left to relax (relaxation phase). Finally, the stage is moved back to its initial position and the bundle recovers its straight configuration. Time is in seconds, bar is 10 µm.

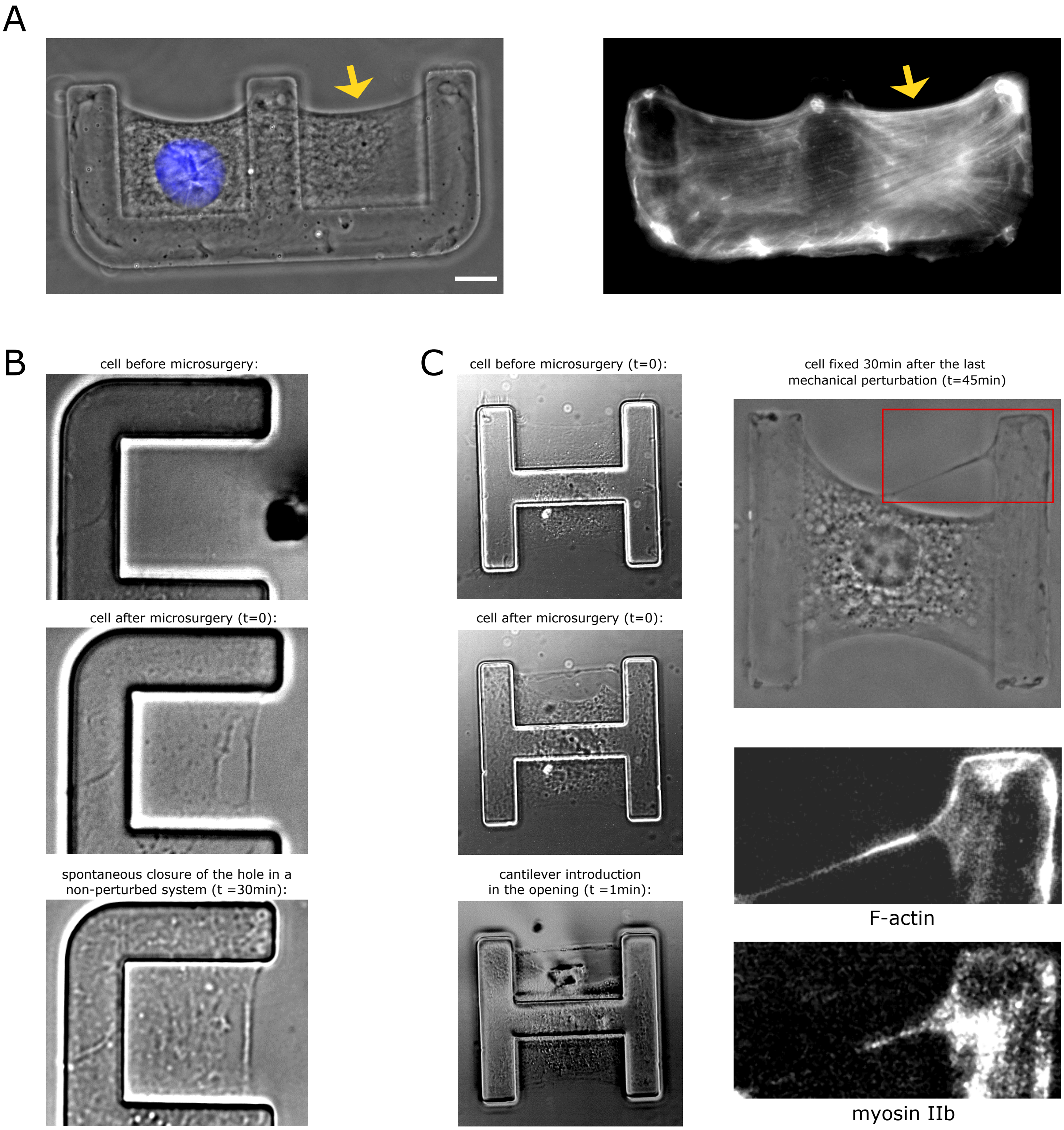

Supplementary Figure 1. Cell morphology before and after microsurgery. A. Brightfield (left) and F-actin staining with phalloidin-AlexaFluor568 (right) of a cell on a E-shape. Yellow arrows indicate a typical region of the cell on the part of the pattern not containing cell nucleus where microsurgery would be performed. Nucleus is shown in blue on the left panel (DAPI). Note the peripheral fiber and numerous supporting fibers. **B.** Sequence of spontaneous closure of the hole in the absence of the cantilever, 30 minutes after micro-surgery. **C.** Sequence of microsurgery, cantilever introduction and initiation of spontaneous closure of the hole through sliding of the fiber towards the cell body. Image in the upper right panel represents the cell fixed with 4% PFA after manipulation. Staining for F-actin and myosin IIb of the region indicated by the red box are shown in the lower panels. Bar is 10 µm: width of the pattern is the same for all the shapes (S-E-H).

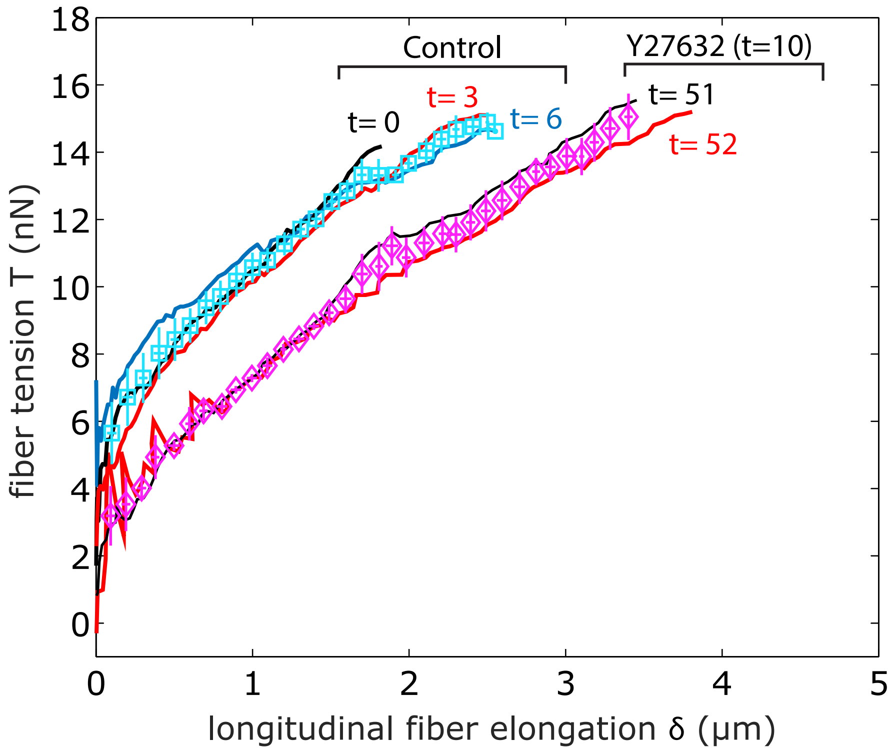

Supplementary Figure 2. Repetitive measurements of tension during the extension phase on the same stress fiber before and after the addition of Y27362. The bundle was subjected to several cycles of variable extension (δ). After each extension, the cantilever was withdrawn and the bundle allowed to recover its straight configuration. “t” values indicate elapsed time from the beginning of the experiments in minutes. Drug was added after 10 minutes from the beginning. Cyan and magenta curves show averages of the measurements before and after treatment respectively. Data points have been grouped by 0.1 µm increments in fiber elongation. Horizontal and vertical errorbars show standard deviation.

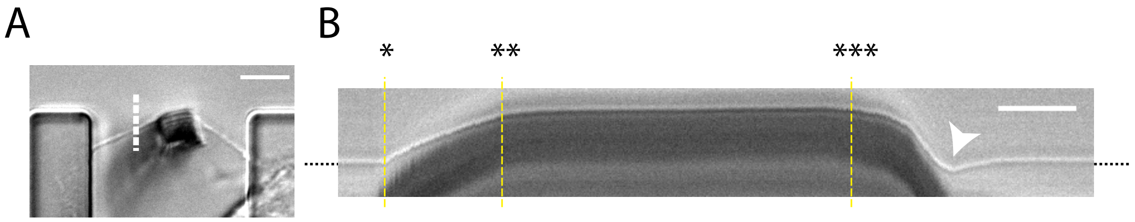

Supplementary Figure 3. Stress fibers maintain visco-elastic properties upon myosin II inhibition. A. Snapshot from a time-lapse of bundle manipulation in a cell treated with Y27632. Bar is 10 µm. **B.** Kymograph along the dashed white line in A, showing bundle displacement. Dashed yellow lines show the different phases of the experiment: (*) beginning of stretching phase, (**) end of stretching phase and beginning of relaxation phase, (***) withdrawal of the cantilever when the bundle begins to retract. The retraction velocity is limited by the cantilever velocity, set to 5 µm/s, as during the stretching phase. The bundle is transiently pulled back farther than its rest position, due to adhesive contact with the cantilever (white arrowhead), and then returns to its initial position indicated by the black dotted line. Bar is 3 s.
